# Balancing Risks versus Benefits: Vitamin C Therapy versus Copper Oxide Nanoparticles Toxicity in Albino Rats' Submandibular Salivary Gland

**DOI:** 10.1055/s-0044-1786867

**Published:** 2024-05-24

**Authors:** Mohamed Shamel, Safaa Baz, Heba Mahmoud, Salma Awad Taghyan, Mahmoud M. Bakr, Mahmoud Al Ankily

**Affiliations:** 1 Department of Oral Biology, Faculty of Dentistry, The British University in Egypt, Cairo, Egypt; 2 Department of Oral Pathology, Faculty of Dentistry, The British University in Egypt, Cairo, Egypt; 3 General Dental Practice, School of Medicine and Dentistry, Griffith University, Gold Coast, Queensland, Australia

**Keywords:** antioxidant, caspase 3, copper oxide nanoparticles, CuO-NPs, K _i_ -67, salivary gland, vitamin C

## Abstract

**Objectives** This study aimed to examine the suppressive effect of the natural antioxidant vitamin C (VC) against submandibular gland toxicity induced by copper oxide nanoparticles (CuO-NPs).

**Materials and Methods** Three groups of 30 mature male albino rats (4 weeks old) weighing between 150 and 200 g were selected. The rats were randomly assigned for 6 weeks to receive: intraperitoneal injection (IP) of vehicle (control group); IP of 2.5 mg/kg body weight (bw) of CuO-NPs (CuO-NPs group); and IP of 2.5 mg/kg bw of CuO-NPs, combined with a daily oral dose of 100 mg/kg bw of VC in drinking water via gavage (CuO-NPs/VC group). The rats were euthanized, and their submandibular glands were dissected for histological evaluation, including hematoxylin and eosin staining and immunohistochemistry for K _i_ -67 and caspase-3.

**Statistical Analysis** The area expression for K _i_ -67 and caspase-3 was statistically analyzed using GraphPad Prism. Following analysis of variance analysis, Tukey's post hoc was used for multiple comparisons. The significance level was set at *p* < 0.05.

**Results** CuO-NPs caused significant cytotoxic effects on submandibular salivary gland cells in albino rats. This led to an increase in K _i_ -67 and caspase-3 levels compared with the control group. VC administration improved tissue histology and reduced K _i_ -67 and caspase-3 levels in the VC/CuO-NPs group compared with rats treated with CuO-NPs alone.

**Conclusion** The study revealed significant cytotoxic effects of CuO-NPs on the submandibular salivary gland of albino rats. VC effectively mitigated these toxic effects, suggesting its potential as a readily available antioxidant.

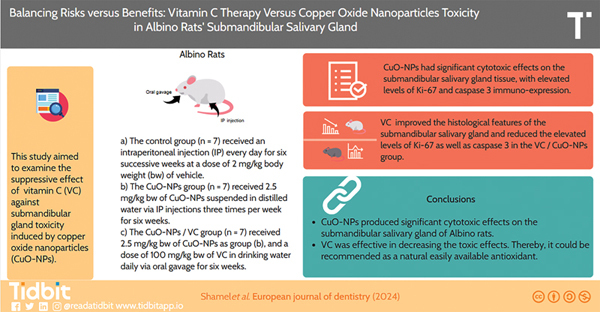

## Introduction

Copper (Cu) has long been acknowledged for its biochemical, anti-inflammatory, and antimicrobial properties. Cu's application in medicine has expanded due to developments in nanotechnology, improving its effectiveness in manufacturing methods. 1 Cu nanoparticles (Cu-NPs) can be synthesized either naturally or through chemical processes. 2

Cu oxide nanoparticles (CuO-NPs) have attracted a lot of interest in medical applications because of their exceptional chemical and physical characteristics, along with their ability to improve end products. These nanoparticles are currently being explored for their potential in various medical fields, such as antimicrobial, anticancer, and drug delivery systems. The high demand for CuO-NPs is driven by their beneficial properties, which not only enhance the overall quality of end products but also contribute to their efficacy in diverse applications. 3 The application of CuO-NPs has shown promising results, particularly in the antimicrobial and anticancer fields, 4 making them valuable for further medical research and development.

The relationship between CuO-NPs and various diseases may emerge with excessive nanoparticle utilization. This is because they can penetrate biological barriers and generate reactive oxygen species (ROS). This may result in cellular damage and oxidative stress (OS). 5 6 Furthermore, increased OS triggered by CuO-NPs activates regulatory pathways, resulting in elevated expression of proinflammatory cytokines and free radical generation. 7 The size of CuO-NPs also plays a crucial role in their toxicity; smaller nanoparticles often exhibit greater toxicity due to their increased surface area and reactivity. 8

Additionally, the accumulation of nano-Cu particles in various organs is reported. 9 Excessive ROS production may disrupt cells' antioxidant defense mechanisms, leading to OS, inflammation, and ultimately cellular damage. 10 Given that antioxidants can neutralize ROS and reduce OS, 11 it is crucial to utilize them to mitigate the toxic effects of CuO-NPs and ensure their safe application.

Vitamin C (VC), a potent antioxidant, is a naturally occurring organic compound that acts as a cofactor and cosubstrate in many cellular processes. It acts as a redox buffer, effectively blocking peroxide-radical-induced lipid peroxidation and neutralizing ROS. Moreover, VC protects cellular components from oxidative damage, thus maintaining cellular integrity and function. 12

This study holds significant importance as it aims to advance the understanding of VC and CuO-NPs, potentially contributing to medical science and health care. By addressing safety concerns surrounding nanoparticle use, this research may pave the way for innovative medical applications while ensuring the well-being of patients and consumers.

Based on the previously mentioned data, this study was designed to evaluate the therapeutic effect of VC versus CuO-NPs-induced salivary gland toxicity in albino rats' submandibular salivary glands to understand its mechanism of action, which may pave the way for its potential therapeutic applications.

## Material and Methods

### Preparation of CuO-NPs

NanoTech Egypt Company provided CuO-NPs. Nanoparticles were suspended in water using sol-gel technology. When the Cu acetate solution was mixed with an aqueous solution of sodium hydroxide (NaOH), a precipitate was formed. 13

### Characterization of CuO-NPs

The synthesized CuO-NPs were carefully characterized by NanoTech Egypt Company to make sure they fulfilled the necessary requirements. The nanoparticles were dark brown colored in powdered form. The solubility testing indicated that they were suspended in water.

NanoTech Egypt Company utilized transmission electron microscopy (TEM) and X-ray diffraction (XRD) analysis for characterization. TEM, using a JEOL JEM-2100 high-resolution TEM at 200 kV, was used to evaluate the nanoparticles' size and shape. The resultant CuO-NPs had an average size of 35 ± 5 nm and a spherical shape. XRD analysis was performed to confirm the structural pattern of the nanoparticles. The results confirmed the nanocrystalline pattern.

### Vitamin C

Vitacid-C tablets were supplied by Chemical Industries Development Company (Giza, Egypt). The tablet contained 1 g of ascorbic acid and was dissolved in water.

### Animals and Experimental Procedures

Twenty-one male albino rats, aged 4 weeks, with weights between 150 and 200 g, were obtained from Animal House at the British University in Egypt (Cairo, Egypt). They were housed in a controlled pathogen-free environment at ± 2°C and a 12-hour dark/light cycle. G*Power, version 3.1.9.2 (University Kiel, Germany), was utilized to calculate the sample size. The effect size was 0.95, with *α* and *β* levels of 0.05, indicating a power of 95%. The estimated number ( *n* ) consisted of 21 samples for the three study groups.

The albino rats were kept in standard individual cages, with five rats per cage. They received standard laboratory feed and water during the study, along with additional treatment specific to each group. Furthermore, the Animal Ethical Committee of the British University in Egypt (approval code 2320, 2023) authorized all research procedures involving animals. The committee ensured compliance with the ethical committee guidelines.

Experimental rats, after 1 week of adaptation, were equally randomized into three groups as follows:

1. The control group ( *n* = 7) received an intraperitoneal injection (IP) every day for 6 successive weeks at a dose of 2 mg/kg body weight (bw) of vehicle nitrate buffer solution. 14
2. The CuO-NPs group ( *n* = 7) received 2.5 mg/kg bw of CuO-NPs suspended in distilled water via IP injection three times per week for 6 weeks, for a total of 18 injections. 15
3. The CuO-NPs/VC group ( *n* = 7) received 2.5 mg/kg bw of CuO-NPs suspended in distilled water via IP injection three times per week for 6 weeks. 15 Additionally, they were administered a dose of 100 mg/kg bw of VC in drinking water daily via oral gavage for 6 weeks. 16

**Fig. 1:**
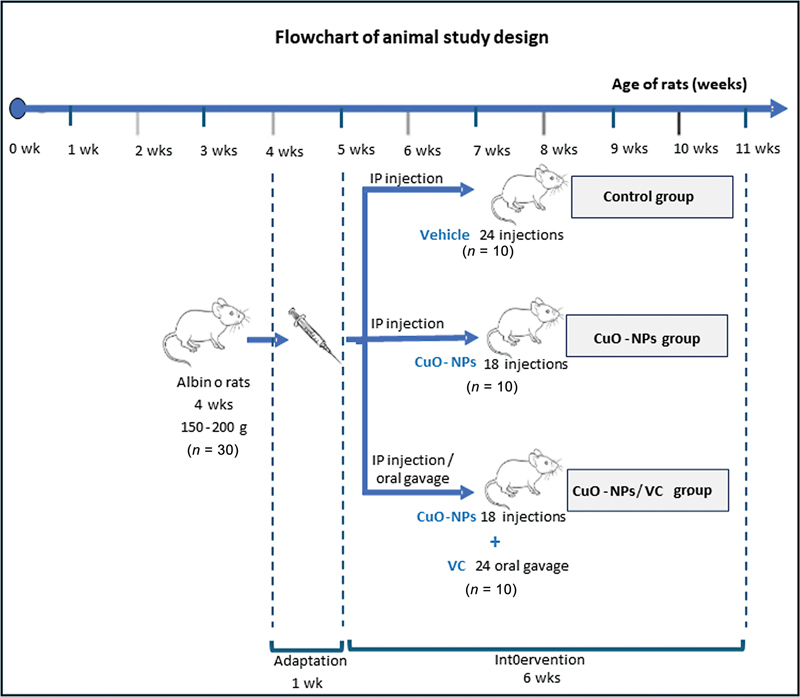
The flowchart of the animal study design.

Ultimately, following the determined experimental period, all rats' major submandibular glands underwent the following steps: dissection, dehydration in ever-increasing grades of alcohol, fixation in 10% buffered formalin, covering in xylene, and paraffin-embedding. Subsequently, sections thickened to 4 to 5 µm were prepared for subsequent hematoxylin and eosin (H&E) and immunohistochemical (IHC) evaluations. The rats were euthanized by receiving overdoses of pentobarbital anesthesia. The flowchart of the animal study design is illustrated in Fig. 1 .

### Histological Analysis

Submandibular salivary gland sections were obtained and fixed in 10% formalin. Following this, the samples underwent serial dehydration with ascending concentrations of ethanol. Then, sections were clarified in xylene and embedded in paraffin. Samples were subjected to H&E staining (5 μm slices). The slides were then examined and photographed under a light microscope (Leica DM 1000, Danaher Corporation, United States).

### Immunohistochemical Analysis for K _i_ -67 and Caspase-3

All IHC staining steps were performed according to the manufacturer's instructions. Briefly, the previously prepared formalin-fixed paraffin-embedded sections (5 μm slices) were mounted on electrically charged slides. The slides were dewaxed and dehydrated, then immersed in an antigen retrieval solution before being blocked with a 5% bovine serum albumin solution. The sections were incubated overnight with anti-K _i_ -67 (#ab16667, Abcam) as well as anti-caspase-3 (#ab184787, Abcam) primary antibodies (1:200 dilutions) at 4°C. After that, the sections were subjected to secondary antibodies and incubated for 2 hours at room temperature. Finally, the solution of the substrate, chromogen, was applied. Slides were then subjected to Harris hematoxylin staining for IHC detection.

### Image Analysis

For IHC analysis, the immunostained slides were evaluated via the image analyzer Leica Qwin 500 software. The expression of caspase-3 and K _i_ -67 was assessed using a light microscope conveyed onto a screen (magnification ×400). This involved determining the proportion of cells with positive immunostaining per a total of 100 cells in 10 fields for each group, which is a standard measurement.

### Statistical Analysis

Statistical analysis for the K _i_ -67 and caspase-3 area expressions was performed using GraphPad Prism (GraphPad 9.0 Software, San Diego, California, United States). At *p* < 0.05, a value for statistical significance was established. Standard deviations and means were used to express the data. For multiple comparisons, Tukey's post hoc analysis was performed after a one-way analysis of variance.

## Results

### Histological Results (H&E-Stained Sections)

**Fig. 2:**
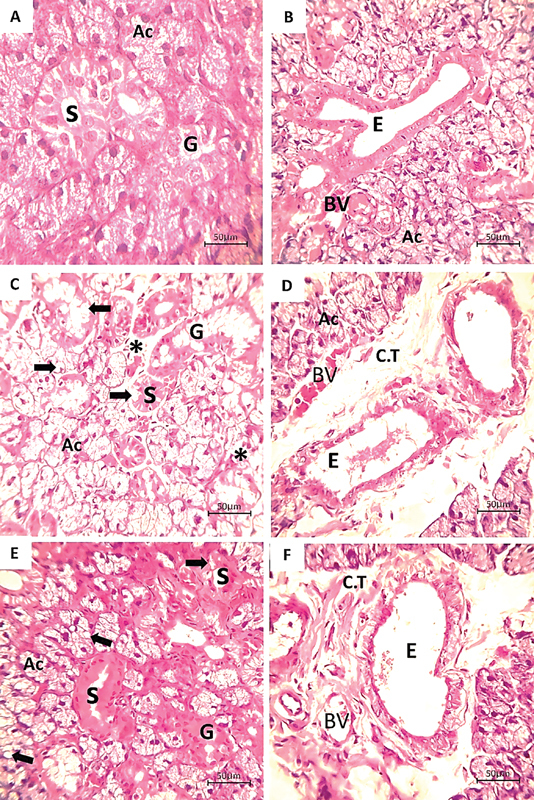
A hematoxylin and eosin (H&E) section of the albino rat submandibular gland. ( **A** , **B** ) The control group showed a normal histology of the gland architecture. ( **C** , **D** ) The copper oxide nanoparticles (CuO-NPs) group showed areas of gland architectural loss with degeneration (asterisk), reduced basophilic cytoplasm of (Ac), eosinophilic granules of (G), and unclear basal striae of (S) with extensive vacuolization (arrows) through the parenchymal component. The (E) was dilated and retained secretion associated with congested (BV) in the C.T. septa. ( **E** , **F** ) The CuO-NPs/vitamin C (CuO-NPs/VC) group showed distinct boundaries of Ac, elevated basophilic cytoplasm of Ac, and eosinophilic granules of G, as well as a decrease in intracytoplasmic vacuolization (arrows) through the parenchymal component. Evident basal striations of (S) and ductal dilatation of (E) with scarce secretion were detected (H&E, original magnification ×400). Ac, serous acinar portions; G, granular convoluted tubule; S, striated duct; E, excretory duct; BV, blood vessel.

*Control group* : The investigation demonstrated typical histological features of the submandibular gland, including both parenchymal and stromal elements ( Fig. 2A and B ).
*CuO-NPs group* : Following CuO-NPs administration, the submandibular salivary gland exhibited several changes. These included large intracytoplasmic vacuolization and reduced intracytoplasmic basophilic staining of the acini. Additionally, areas of gland architectural loss with degeneration were observed. Granular cell tumors (GCTs) had vacuolization and a decrease in eosinophilic granules in the duct system. The striated ducts showed vacuolations and unclear basal striae ( Fig. 2C ). Numerous blood vessels were congested, and some excretory ducts were dilated with retained secretion ( Fig. 2D ).
*Combined CuO-NPs/VC group* : When compared with the CuO-NPs group, VC treatment improved the glandular architecture. The intracytoplasmic vacuolations throughout the gland were noticeably reduced. Additionally, the cytoplasmic basophilia of the acini was enhanced, and their boundaries became more clearly defined as a result. The GCTs' eosinophilic granular content increased in the duct system ( Fig. 2E ). The prominent basal striations of the striated duct were apparent. The excretory ducts in the connective tissue septa hardly retained the secretion associated with dilated blood vessels ( Fig. 2F ).

### Immunohistochemical Results

#### Immunohistochemical Expression of the K _i_ -67 Antibody

**Fig. 3:**
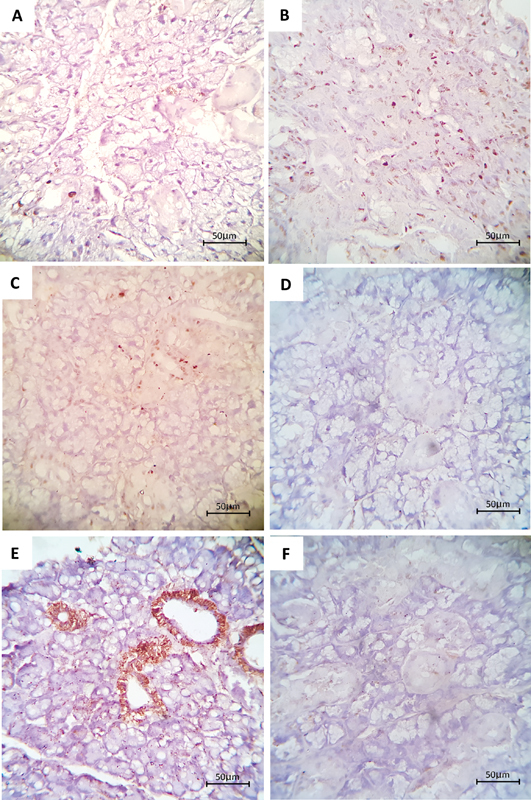
An immunostained section of anti-K _i_ -67 and caspase-3 antibodies of the albino rat submandibular gland. ( **A** , **D** ) The control group showed nuclear and cytoplasmic immunoreactivity among the whole glandular parenchyma for K _i_ -67 and caspase-3, respectively (mild to moderate immunoreactivity). ( **B** , **E** ) The copper oxide nanoparticles (CuO-NPs) group showed positive nuclear expression of K _i_ -67 throughout the gland, and caspase-3 immunoreactivity presented a diffuse expression with a moderate cytoplasmic reaction in acinar cells and a strong positive reaction in the ductal cells. ( **C** , **F** ) The CuO-NPs/vitamin C ( CuO-NPs/VC) group showed positive nuclear and cytoplasmic immunoreactivity among the ductal cells for K _i_ -67 and caspase-3, respectively (mild to moderate immunoreactivity) (Diaminobenzidine (DAB), original magnification ×400).

*Control group* : The evaluation revealed a few nuclear-positive K _i_ -67 immunoreactions throughout the gland. The stained nuclei showed mild to moderate immunoreactivity for K _i_ -67 ( Fig. 3A ).
*CuO-NPs group* : The examination revealed an obvious increase in the percentage of positive K _i_ -67 expression in comparison to the control group. This increase was observed within the nuclei of the parenchymal element. The stained nuclei showed moderate to strong immunoreactivity for K _i_ -67 ( Fig. 3B ).
*Combined CuO-NPs/VC group* : In comparison to the CuO-NPs group, the examination of the CuO-NPs/VC group showed an apparent reduction in the percentage of positive K _i_ -67 expression. This reduction was observed within the nuclei of the parenchymal element. The stained nuclei showed mild to moderate immunoreactivity for K _i_ -67 ( Fig. 3C ).

#### Immunohistochemical Expression of the Caspase-3 Antibody

*Control group* : The control group's rat submandibular salivary gland exhibited positive staining with the anti-caspase-3 monoclonal antibody. This staining reaction was observed in the cytoplasm of the whole glandular component. The immunoreactivity against caspase-3 was mild to moderate ( Fig. 3D ).
*CuO-NPs group* : Examining the sections of the rat submandibular salivary gland in the CuO-NPs group revealed an apparent increase in caspase-3 expression. This increase was observed in both the acini and ducts compared with the control group. In the acini, the cytoplasmic immunostaining ranged from moderate to strong, whereas the ductal cells displayed strong cytoplasmic immunoreactivity ( Fig. 3E ).
*Combined CuO-NPs/VC group* : It was evident that the CuO-NPs/VC group had mild to moderate cytoplasmic immunoreactivity to caspase-3. This immunoreactivity showed fewer positive expressions throughout the entire parenchymal element compared with the CuO-NPs group ( Fig. 3F ).

### Statistical Results

**Fig. 4:**
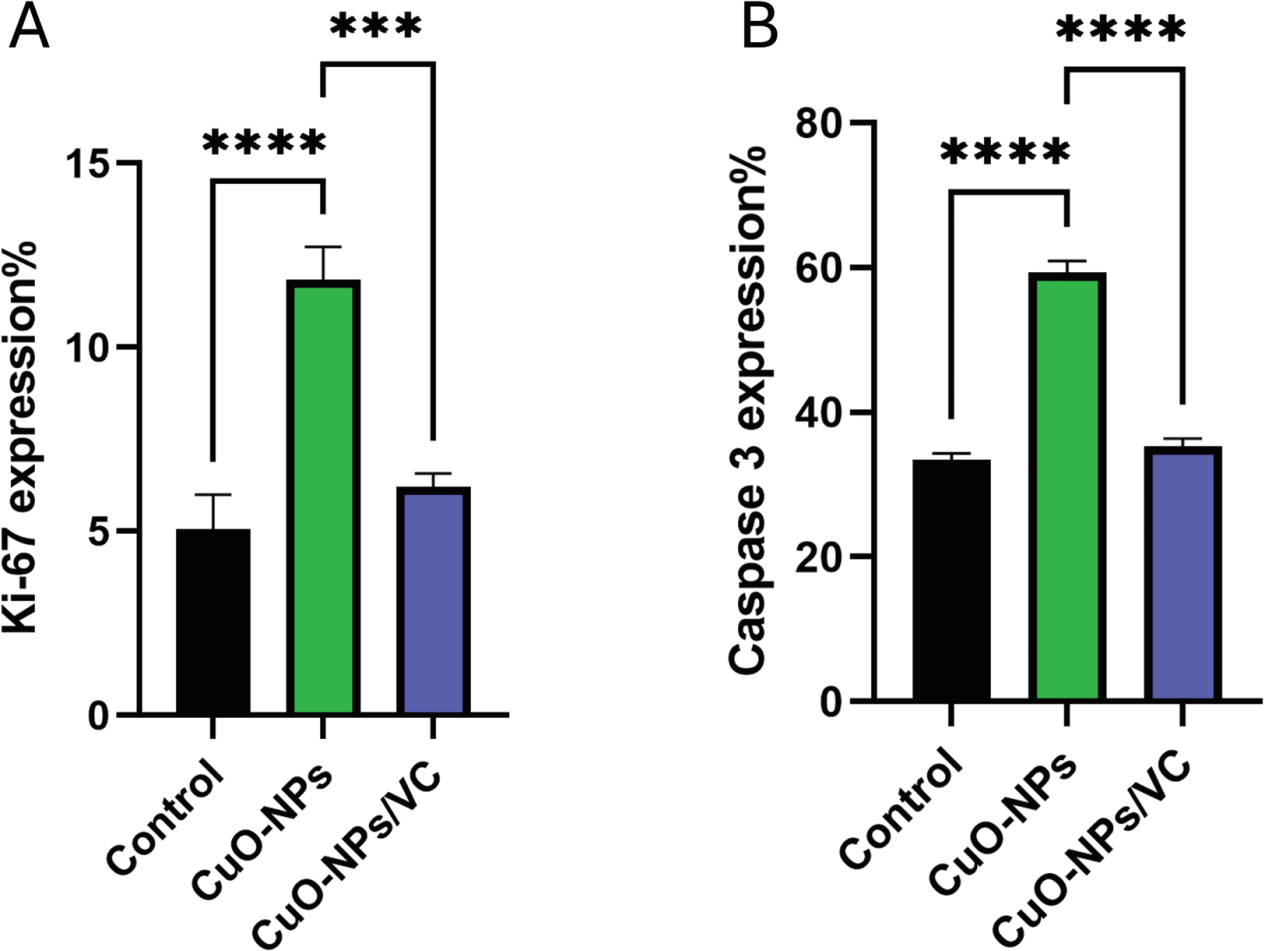
Bar charts representing mean area percentage in different groups. ( **A** ) K _i_ -67 expression percentage. ( **B** ) Caspase-3 expression percentage. **** *p* < 0.0001 and *** *p* < 0.001 for each group in relation to the control group and group-to-group variations.

The CuO-NPs group had the highest mean area percentage of K _i_ -67 immunoexpression, while the control group had the lowest value. The post hoc Tukey test revealed a significant statistical difference in K _i_ -67 area% expression between the CuO-NPs group and the control group ( *p* < 0.0001). Furthermore, there was a statistically significant difference ( *p* = 0.0003) between the CuO-NPs group and the CuO-NPs/VC group. Nevertheless, there was no statistically significant difference ( *p* = 0.2445) between the CuO-NPs/VC group and the control group ( Table 1 ) ( Fig. 4A ).

**Table 1:** Tukey's post hoc test for K _i_ -67 area expression in different groups

Tukey's multiple comparisons test	Mean difference	95% CI of the difference	*p* -Value
**Control group versus CuO-NPs group**	–6.777	–8.720 to –4.834	< 0.0001 a
**Control group versus CuO-NPs/VC group**	–1.147	–3.090 to 0.7963	0.2445
**CuO-NPs group versus CuO-NPs/VC group**	5.630	3.687 to 7.573	0.0003 a

Abbreviations: CuO-NPs, copper oxide nanoparticles; VC, vitamin C.

Note: One-way analysis of variance (ANOVA) analysis of K _i_ -67 expression.

a Indicates significance.

Similarly, the caspase-3 immunoexpression indicated that the CuO-NPs group presented the greatest mean area% of the rat submandibular salivary gland. The control group had the lowest value. In comparisons between different groups, the post hoc Tukey test revealed statistically significant differences in caspase-3 expression. Specifically, there were significant differences between the CuO-NPs group and the control group ( *p* < 0.0001), as well as between the CuO-NPs group and the CuO-NPs/VC group ( *p* < 0.0001). However, no significant difference was observed between the CuO-NPs/VC group and the control group ( *p* = 0.2165) ( Table 2 ) ( Fig. 4B ).

**Table 2:** Tukey's post hoc test for caspase-3 expression in different groups

Tukey's multiple comparisons test	Mean difference	95% CI of the difference	*p* -Value
**Control group versus CuO-NPs group**	–25.87	–28.95 to –22.79	< 0.0001 a
**Control group versus CuO-NPs/VC group**	–1.917	–4.998 to 1.165	0.2165
**CuO-NPs group versus CuO-NPs/VC group**	23.95	20.87 to 27.03	< 0.0001 a

Abbreviations: CuO-NPs, copper oxide nanoparticles; VC, vitamin C.

Note: One-way analysis of variance (ANOVA) analysis of caspase-3 area expression.

a Indicates significance.

## Discussion

Despite the wide applications of nanotechnology, including CuO-NPs, there are many undesirable concerns about human health. Therefore, nowadays, CuO-NPs have been highlighted as an attractive era for research. 17

In the current study, IP injections were chosen as the route of administration of CuO-NPs because they are more precise, dependable, and repeatable. This ensures that rats consistently receive the same CuO-NPs dosage, minimizing the risk of subchronic toxicity compared with other exposure routes. 15 Therefore, this research aimed to assess VC's potential therapeutic impact on the altered salivary glands caused by CuO-NPs.

In this study, the histology of the control group rats' submandibular salivary glands showed normal characteristics of the salivary gland architecture. However, the CuO-NPs group had several changes, including massive intracytoplasmic vacuolization and reduced cytoplasmic basophilia of the acini. Accordingly, previous studies reported that cellular vacuolization indicates the development of diseased tissue rather than the regeneration process. This could result from lipid droplet accumulation due to the reduced activity of the utilized fatty acids. 18 19

Moreover, this vacuolization and loss of basal striations could be attributed to mitochondrial damage. Mitochondria are highly susceptible to toxic agents that allow the entry of sodium ions, leading to the failure of cellular metabolism. Furthermore, it was reported that the cytoplasmic vacuoles could be formed because of osmotic pressure, with a consequent breakdown of large macromolecules within the injured cell. 20

In the same context, another study with similar results attributed this vacuolization to zinc oxide nanoparticles inducing toxicity. This toxicity leads to OS and mitochondrial dysfunction, ultimately reducing the available cellular energy. 14 This could also explain the observed stagnated secretion in the excretory duct.

The signs of degeneration in acinar and ductal cells could be related to the potential genotoxic effects of CuO-NPs. Ghonimi et al demonstrated that CuO-NPs had a toxic impact on hepatic and renal tissues, supporting this possibility. 21 Additionally, a previous study that evaluated the impact of oral exposure to CuO-NPs on the rats' liver revealed significant hepatotoxicity in OS. 22 This CuO-NPs' toxicity might return to the production of cellular ROS. This leads to the breakdown of deoxyribonucleic acid (DNA) strands, subsequently altering gene expression. 23 Similarly, it was reported that the creation of ROS induces DNA damage that initiates signaling pathways and ultimately results in cell death. 24

VC was chosen as the antioxidant in the present study due to its potent capacity to eliminate free radicals and counteract OS as a powerful antioxidant. 25 VC plays diverse protective roles in pathological conditions and at the OS level. 26

The dose of VC used in this investigation was selected as it was previously shown to be effective in reducing OS and lipid peroxides. Thereby, VC could counteract the toxicity of lead nanoparticles. 16

The submandibular salivary tissues of the CuO-NPs/VC group in the present research had nearly more regular histological features in comparison to the CuO-NPs group. VC could decrease the toxic effect of CuO-NPs in the submandibular salivary glands of rats. This may be related to the decline in ROS generation that results in toxicity. The intake of VC reduces the level of lipid peroxide generated by OS. This is due to the existence of a membrane rich in oxidizable polyunsaturated fatty acids. 27 The antioxidant agents can limit the production of ROS and reduce unfolded protein reactions and protein misfolding. This could explain the increased acinar basophilia found in the current study. 28

The K _i_ -67 nuclear marker plays a crucial role in cell proliferation regulation throughout the cell cycle. It is commonly used to detect neoplastic growth and is associated with cell cycle progression. Besides its proliferative index, K _i_ -67 has a crucial role in DNA repair mechanisms. 29

Concerning the K _i_ -67 IHC results, the control group demonstrated mild nuclear immunoreaction across a small number of nuclei in rat submandibular gland tissue, while the CuO-NPs group had a higher level of K _i_ -67 in comparison to the control group. Consistent with a study by Taha et al, which investigated the cytotoxicity of silver nanoparticles on rats' parotid glands, their IHC results showed intense nuclear K _i_ -67 immunoreactivity in both acinar and ductal cells. However, they found K _i_ -67 immunoreactivity within the cytoplasm of these cells. 30 A study performed by Anreddy in 2018 attributed the increase in the immunoexpression of K _i_ -67 to the toxicity of CuO-NPs. This toxicity resulted from OS due to the production of ROS and cellular metabolic imbalance. 22

In the CuO-NPs/VC group, the nuclear K _i_ -67 immunoexpression was significantly decreased. This could be attributed to the antioxidant activity of VC by increasing antioxidant enzymes and other components to overcome the cytotoxicity of the metallic nanoparticles. 31

Caspase proteins are key regulators of apoptosis, either through intrinsic or extrinsic mechanisms. Caspase-3 plays a role in both pathways. 32 In this study, active caspase-3 staining was used to investigate and detect apoptosis of the submandibular salivary glands. CuO-NPs were reported to induce apoptosis through mitochondrial dysfunction, a reduction in adenosine triphosphate levels, and an elevation in the activity of the caspase-3 enzyme. 33

From the IHC results, an increase in caspase-3 expression was observed in the CuO-NPs group. This increase was observed within the parenchymal elements of the gland in comparison with the control group. A statistically significant difference between the two groups was recorded ( *p* < 0.0001). The results endorsed the role of CuO-NPs-induced apoptosis in the death of the glandular cells.

A previous study performed by Ghonimi et al determined the cytotoxic effects of CuO-NPs and the possible mechanism of apoptosis. They reported the association of apoptosis with upregulation of caspase-3 protein immunoexpression in different tissues. 21

This aligns with a study by Dey et al. They analyzed the toxicity of CuO-NPs and their ability to trigger apoptosis in cancer cells. They reported an increase in caspase-3 protein expression with statistical significance compared with the normal control group. Noteworthy, the apoptotic events of CuO-NPs could be utilized in cancer treatment. This occurs through the successful cellular uptake of Cu ions, resulting in the generation of ROS and nuclear fragmentation, with consequent DNA damage. 34

The gland components in the CuO-NPs group showed moderate reactivity to the apoptotic protein marker caspase-3, with ductal elements staining stronger than acinar ones. The diffuse IHC expression pattern suggests apoptosis affecting both acinar and ductal cells, possibly due to mitochondrial dysfunction and activated caspase-3 enzymatic activity. The concentration difference may be due to extensive basal membrane infoldings in the striated ducts, rich in mitochondria, hinting at apoptosis via a mitochondria-mediated pathway. 35 36 However, this only explains the strong staining in striated ducts.

In the VC/CuO-NPs-treated group, there was mild to moderate caspase-3 positivity in parenchymal elements. This might be explained by VC's antioxidant properties, neutralizing CuO-NPs-induced ROS and preventing lipid oxidation and mitochondrial permeability, thus defending against OS. 25 37 Additionally, VC's cellular antiapoptotic effect could contribute to this. However, it contrasts with findings by Zhou et al, who observed VC promoting apoptosis in oral cancer cell lines by increasing ROS levels, leading to DNA breakdown and modulation of Bcl-2, Bax, and caspase-3 expression. 38

The statistical findings validated the IHC analysis. The CuO-NPs group exhibited a significant elevation in caspase-3 expression compared with the control group. Regarding the VC/CuO-NPs group, caspase-3 immunoexpression was significantly downregulated compared with the CuO-NPs group.

In the VC/CuO-NPs group, VC inhibited the elevation of K _i_ -67- and caspase-3-positive cells. This suggests that VC has a notable therapeutic effect on OS and DNA damage caused by CuO-NPs. This inhibition reduced cellular proliferation and increased apoptosis in the rat submandibular gland. However, the underlying process by which OS could influence proliferation and other cellular alterations remains unclear. Vital et al supported the theory that this is due to two separate mechanisms. First, it is well recognized that OS can increase cell proliferation. This occurs by activating specific signaling pathways, such as mitogen-activated protein kinases and phosphatidylinositol 3-kinase/protein kinase B. Second, oxidative DNA damage may induce cell cycle arrest with the secondary secretion of numerous cytokines. 39

## Conclusion

In conclusion, CuO-NPs have potential cytotoxic effects on the parenchymal elements of the submandibular gland. Additionally, this study indicated that VC was highly effective in diminishing such toxicity; thereby, it might be suggested as a readily available, natural source of antioxidants. In addition, more clinical trials could be performed to investigate other possible clinical applications of VC.
